# Neglected Value of Small Population-based Surveys: A Comparison with Demographic and Health Survey Data

**Published:** 2015-03

**Authors:** Anne C. Langston, Debra M. Prosnitz, Eric G. Sarriot

**Affiliations:** ^1^International Rescue Committee, New York, NY, USA; ^2^ICF International, Washington, DC, USA; ^3^ICF International, Center for Design and Research in Sustainable Health and Human Development (CEDARS), Rockville, MD, USA

**Keywords:** Child health, Methodology, Mortality, Survey

## Abstract

We believe that global health practice and evaluation operate with misleading assumptions about lack of reliability of small population-based health surveys (district level and below), leading managers and decision-makers to under-use this valuable information and programmatic tool and to rely on health information from large national surveys when neither timing nor available data meet their needs. This paper uses a unique opportunity for comparison between a knowledge, practice, and coverage (KPC) household survey and Rwanda Demographic and Health Survey (RDHS) carried out in overlapping timeframes to disprove these enduring suspicions. Our analysis shows that the KPC provides coverage estimates consistent with the RDHS estimates for the same geographic areas. We discuss cases of divergence between estimates. Application of the Lives Saved Tool to the KPC results also yields child mortality estimates comparable with DHS-measured mortality. We draw three main lessons from the study and conclude with recommendations for challenging unfounded assumptions against the value of small household coverage surveys, which can be a key resource in the arsenal of local health programmers.

## INTRODUCTION

Providing information to local health planners, implementers, and donors about the trends in health indicators they are trying to improve is crucial for evidence-based programming and appropriate management responses. While service statistics are essential for monitoring service delivery, quality, continuity and can provide some information on reach of services, population-based health surveys are essential to estimate population health status, evaluate progress over time as well as provide a range of socioeconomic correlates, especially in low- and middle-income settings where governmental HMIS and vital registration are not adequate.

We compare the use of small-to-mid-size population surveys with large national-to-regional health surveys, such as the Demographic and Health Surveys (DHS) and Multiple Indicator Cluster Surveys (MICS). By small-to-mid-size surveys, we refer to population surveys representative of subdistrict, district, and, occasionally, multidistrict populations opposed to surveys providing national to regional/provincial estimates. We believe that global health practice underestimates the value and efficiency of small population health surveys and that managers and decision-makers are too often forced to rely on health information from large national surveys when neither timing nor geographic disaggregation of data meets their needs. The reliability of small population-based surveys has long been tested and documented, either using cluster sampling or stratified direct random sampling [i.e. Lot Quality Assurance Sampling (LQAS)] (1–3). In day-to-day practice in global health, however, the value of their data is commonly questioned or overlooked. Donors tend to question the technical rigor and, thus, validity of surveys done by organizations other than universities or research firms, such as NGOs, despite the demonstrated capacity of many. Further, monitoring and evaluation activities have historically been a low priority for donors and governments, although this trend is changing. Small population-based surveys are consequently grossly underutilized by implementers and researchers. “At this level (district), all we have to use is health service data,” is the misleading maxim, which still dominates technical discussions in health information, monitoring and evaluation circles. This practice bias about small population surveys can be illustrated by their absence from guiding documents on the monitoring and evaluation of health programmes (4).

Our objective is not to oppose small surveys to large national surveys, which are a basis of essential information for national and global health programmes and policy-makers, neither do we ignore the limitations of small surveys (see ‘Discussion’). We report on a unique opportunity for a real-life comparison opposed to computer simulation (5) between a knowledge, practice and coverage (KPC) survey and data from Rwanda DHS (RDHS) to disprove the suspicions against small surveys and then discuss when and how small surveys should be considered to have a high value for health information at multidistrict to subdistrict levels.

### Population surveys and the level of management response

DHS and MICS (6) represent high standards for health data and can be used for assessing global and national trends in health but rarely below national and regional levels. They offer comparability across regions and countries and high confidence levels for difficult measures, such as mortality indicators. Additionally, these allow a deep and wide database for countless studies on health determinants and drivers of change in population health. Each level of disaggregation allowed by a national survey (regional, province, or district) comes with additional cost**s**, and DHS or MICS usually stops at regional level, with some exceptions.

In the context of health system decentralization and possibly localization (7), programme orientations and management decisions lie increasingly at the district level. Non-governmental organizations (NGOs), bilateral or multilateral projects can and often intervene at the national or policy levels but predominantly work with districts in implementation or technical assistance. At this level, service statistics are an important tool but are widely insufficient to inform district managers on population health status and intervention coverage, and this information gap cannot be bridged by reports of regional averages. This leads to tremendous challenges in providing monitoring signals that may indicate successes or need for change or improvement for interventions at the district level. Projects, sometimes, try to attach themselves to DHS documents to report on progress on health outcomes but this has rarely worked in our experience due to timing of both surveys and survey reports as well as the level of data disaggregation available.

To be most useful, indicators need to be measured at the operational level in ways that are inexpensive and adapted to specific programme needs. Small population surveys can be used at the subnational level to capture baseline data and measure and report on progress specific to their project area (1,8,9). We have repeatedly seen district managers more ready to energize their staff when they are provided with information relevant to their level of decision-making. As initiatives for outreach, quality improvement, or innovations happen with timeframes based on partnership agreements and access to resources, small population surveys also allow pre-post measures on an as-needed basis.

Public health managers are too often working ‘blindfolded’ regarding population health status: “We're waiting for the results of the last National Health Survey for our Province but we really don't know how we compare to the Province as a whole really. It's difficult to set priorities and even more difficult to know if we've been successful.” (1) The knowledge, practice and coverage survey (KPC) was developed to enable programme managers and local authorities to make decisions based on contextually meaningful information.

The KPC is an example of a small population survey method used in estimating levels of and changes in many standard maternal and child health indicators (9). The KPC tool consists of seven modules aligned with technical areas (e.g. sick child and immunization), from which implementing organizations can choose; each contains a questionnaire, key indicators, tabulation plans, and instructions. It is implemented using either a cluster or stratified sample design. The KPC is a tool and process designed to promote local participation in identifying health priorities, ownership of data at local levels, and the use of data for decision-making at local levels. It became a requirement for USAID-funded child survival programmes in 1991 and has since improved the ability of projects to identify priorities, use data to define objectives, and measure progress towards objectives (10).

We frame our study and discussion by comparing KPC and DHS data as illustrations. Additionally, recent work using the Lives Saved Tool (LiST) has shown how the results of KPC surveys can be used for providing estimated child mortality rates and change in child mortality over time. (11)

Our study addresses the first obstacle to recommending or implementing KPC surveys in the field: the never written but often-stated assumption that KPC data (small surveys in general) are not reliable and lack quality.

### Background

One project, which used a KPC survey as a baseline and endline measure of progress, provided the first data elements for our study.

Kabeho Mwana was a USAID-funded child survival project implemented in six of the 30 districts of Rwanda (Gisagara, Kirehe, Ngoma, Nyamagabe, Nyamasheke, and Nyaruguru), covering approximately one-fifth of the population. It was implemented by a consortium of three NGOs led by Concern Worldwide Inc., in partnership with the International Rescue Committee and World Relief. The primary intervention was to train and equip community health workers (CHWs) to provide treatment of diarrhoea, malaria, and pneumonia in the community, known as integrated community case management (iCCM). These CHWs, called *binômes*, are an unsalaried cadre under the MOH's Community Health Desk. Supervision was provided by a Community Health-in-Charge at the government health facilities in the catchment area with support from Kabeho Mwana staff. Kabeho Mwana was the lead initiative supporting CCM in the six districts and a significant contributor to national scale-up (12).

Kabeho Mwana conducted a KPC survey in June and July 2011 to measure changes in key project indicators since the baseline in 2007. The questionnaire was based on the 2006 Rapid Core Assessment Tool on Child Health, which has questions and indicators closely aligned to the DHS (13).

The 2010 Rwanda DHS was implemented by the National Institute of Statistics of Rwanda in collaboration with the Ministry of Health (MOH) and support from ICF Macro with USAID funding to produce national and regional estimates. The fieldwork was conducted from September 2010 to March 2011 (14).

**Table 1.** Characteristics of RDHS and KPC survey

Survey	Dates of data collection	Methodology	Sample-size (project/6 district area)	Responsible agency
RDHS	26 September 2010 to 10 March 2011	Two-stage stratified sampling of villages, random sampling of 26 households per village, all women in household interviewed, and all children included	1,427 households with children aged below 5 years 1,780 living children aged below 5 years	National Institute of Statistics of Rwanda, ICF International
KPC	20 June to 8 July 2011	Two-stage stratified sampling of village and starting household with parallel sampling of subpopulations. Only households with children aged below 2 and/or 5 years were interviewed; only one child under 2 years and/or one recently-ill child aged below 5 years per household	120 households with living children aged below 2 years 120 children aged below 2 years 395 children aged below 5 years ill in the past 2 weeks	Kabeho Mwana Project

## MATERIALS AND METHODS

We compared indicator estimates from the 2010 RDHS and 2011 KPC surveys. Table 1 summarizes the characteristics of the two surveys, which occurred less than a year apart. Although the DHS was designed primarily to produce national and regional estimates, the 2010 RDHS enumeration areas were stratified at the district level. This allowed matching geographic areas between the two surveys and yielded enough power for RDHS estimates for the six districts supported by Kabeho Mwana.

### Method of the KPC survey

*Sampling:* A stratified sample design was used, with a sample-size of at least 100 for all of the key programme indicators, allowing for calculation of the point estimates with a 95% confidence interval of no more than ±10%, depending on the indicator. A secondary objective of the sampling strategy was to evaluate variation between districts and identify any significantly low-performing districts using LQAS. For this purpose, a minimum sample-size of 19 per district was required. A total of 20 villages per district were selected for a sample-size of 120 households with living children below 2 years and 395 children under 5 years, who were ill in the two weeks prior to the survey.

Twenty villages per district were first selected with probability-proportional-to-size using lists of all villages in the six districts, along with their population-size obtained from the National Institute of Statistics. Chiefs of the selected villages were then contacted to obtain a complete listing of all households. From that list, a single household was selected as a starting point, using a random number between one and the total number of households in the village.

To get enough power for analysis of key indicators, data-collection teams carried out parallel sampling (15) of the following two groups:

- Mothers of a child aged 0-23 month(s), who were asked questions on maternal health and health promotion measures, such as breastfeeding, bednet usage, and vaccinations
- Mothers of a child aged 0-59 month(s), who had been sick with any of three conditions of interest in the last two weeks.

Interviewers first visited the household selected at random. Here, they looked for eligible participants; if none was found, they continued to the next closest household. Only one of each type of interview was conducted in a household but information on multiple conditions could be collected for each sick child. If more than one child was found to meet the criteria in the household, a coin was flipped to choose which would be used for data collection. After completing the questionnaire in a household, the team proceeded to the nearest household to complete the remaining quota of interviews. Interviews were conducted in one to four households per village, depending on the number of conditions of interest found in each household. A more detailed description of the survey methodology, tools, and sampling frame is available as an annex to the Kabeho Mwana final evaluation report and upon request. Information was collected on a total of 120 children aged 0-23 month(s) and 395 children aged 0-59 month(s), who had been sick in the last two weeks.

*Implementation*: The survey received approval from the Rwanda National Ethics Committee prior to beginning of data collection.

Ten data-collection teams were formed, with one project staff member and one representative from the Ministry of Health. At least one member of each data-collection team had prior experience on administering surveys. On average, it took a total of two days for five teams to visit the 20 selected villages in each district. Team members worked outside of their usual area of responsibility to minimize conflicts of interest.

Leaders of the survey team received a two-day training on the indicators, survey principles, the rights of participants, the importance of obtaining written informed consent, and data-collection instruments and procedures before team formation. Team leaders, supervisors, and enumerators all participated in a three-and-a-half-day training, including detailed instructions on sampling, interviewing techniques, and practice with and field testing of the questionnaire and sampling methods.

There was one supervisor for every two data-collection teams, allowing them to visit selected villages regularly to verify adherence to the sampling protocol and observe data-collection. They were required to observe at least one interview per team per day and verify every data-collection form prior to the team's departure from the area.

*Data-entry, cleaning, and analysis:* Data-entry was done using a Microsoft Access database. The data were double-entered and checked for consistency. A number of validity checks were run as the first step in the analysis, with inconsistent answers verified using the paper forms. To the village level, the sample was self-weighting. During analysis, weights applied to account for the chance of a given household within the village being selected as the number of households per village varied considerably. When information about more than one of the three conditions of interest was collected in a village, weights were adjusted so that all cases of the condition in the village added up to one. All weighting was applied during the analysis, using the STATA survey functions.(16)

### Method of the RDHS

Consistent with the standard DHS methodology, the 2010 RDHS used a stratified cluster-sampling strategy (17). The 2010 RDHS used the preparatory frame for the Rwanda General Population and Housing Census provided by the National Institute of Statistics of Rwanda. This sampling frame is a complete list of natural villages covering the entire country. From this, 492 villages were selected and stratified by district, after which a complete listing of all households in each village was made. From this list, 26 households per village were randomly selected. The sets of questionnaire used were adapted from the DHS standard questionnaire.

The coverage indicators for the project area were obtained from RDHS data available from the Measure DHS website (http://www.measuredhs.com/Data), using recode documentation and processed using STATA (version 11) (16). Standard weights were applied.

### Lives Saved Tool Modelling Method

Lives Saved Tool (LiST) is a cohort model of child survival from 0 to 59 month(s) of age that provides estimates of child mortality based on the standard child health indicators. It can also provide estimated child mortality rates at the intervention- or district-level, based on data from intervention area through surveys (i.e. KPC), which are not powered to provide direct mortality estimates. Version 4.48 of the LiST, updated in July 2012, was used for modelling. The software and Rwanda trend file were downloaded from the Johns Hopkins Institute for International Programs web site (18). The development of LiST, its structure, and assumptions are described elsewhere (19). It has been previously validated against Demographic and Health Surveys (DHS) (11).

*Data used for modelling in LiST:* The Kabeho Mwana KPC survey collected population coverage data on 14 LiST interventions, nine of which had significant increases over the life of the project. When child health indicators included in LiST were not available for the project area or there was no significant change according to the KPC survey, corresponding national indicators for rural areas from the RDHS were used in the model. LiST estimates the value of nine other indicators from available data (e.g. LiST estimates coverage for syphilis screening from antenatal coverage). Data from two phases of the KPC, at project baseline and endline, were used. The KPC and RDHS data used for LiST modelling are summarized in Table 2.

The effect-sizes used for all interventions were those already included in LiST by the Child Health Epidemiology Reference Group (20). The model was built on the LiST trend file for Rwanda (18). The trend file includes the national cause of death profile, population structure, and fertility data from 2008. The background health status was adjusted to indicate the population as both vitamin A- and zinc-deficient and, per Rwanda's national policy, this indicates that intermittent preventive treatment for malaria in pregnancy (IPTp) is not being implemented.

The LiST model was run using under-five, infant, and neonatal mortality rates re-analyzed from the 2007-2008 Rwanda DHS for the Kabeho Mwana project area only (as described above).

### Comparison of small (KPC) and large (DHS) population-based survey data

The sets of Kabeho Mwana KPC questionnaire were designed to collect a limited set of indicators relating to child health (Table 3). For each of these indicators, equivalents were sought in the RDHS data for the same six districts. Table 3 compares the indicators and explains why several indicators were excluded from the analysis. In a few instances, the questions were simply not asked in RDHS 2010; in others, the definitions or screening questions used could not be reconciled. At the end, 15 out of 19 indicators collected in the KPC were matched with equivalent data from RDHS 2010. We used a 2-tailed *t*-test of the difference between means of KPC and RDHS estimates with alpha=0.05 to determine whether the results were significantly different.

### Comparing mortality estimates as predicted by LiST and as measured by DHS

With coverage inputs from RDHS and the project's baseline and endline KPC surveys as described above, LiST produced an estimate of the under-five mortality rate for comparison to that measured in the project area by the RDHS for the same period.

**Table 2.** Data modelled in LiST

Outcome indicator	Baseline %	Endline %	Source
Skilled birth attendance**	39.0	90.7	KPC
Postnatal visit	13.0	58.0	KPC
Handwashing	2.0	18.6	KPC
Point-of-use water treatment	31.0	64.7	KPC
Vitamin A supplementation	66.0	85.8	KPC
DPT3 coverage	81.0	97.0	KPC
Zinc for diarrhoea treatment	5.0	22.0	KPC
Treatment for malaria	20.0	43.0	KPC
Antibiotics for pneumonia	12.7	63.0	KPC
Antenatal care	23.5	34.7	DHS
Tetanus toxoid vaccination	30.6	33.7	DHS
Iron folate 90+	40.7	73.1	DHS
Exclusive breastfeeding [0-5 month(s)]†	87.1*	84.9	DHS
Piped water	0.9	1.4	DHS
Improved latrine	40.2*	71.9	DHS
ITN-use	31.9*	69.6	DHS
Measles vaccination	90.2	94.8	DHS
Polio (3rd dose)	89.1	93.3	DHS
BCG	95.0	99.1	DHS
Treatment for diarrhoea (ORT)	28.8	34.9	DHS

*Interpolated from 2005 value

**SBA cannot exceed facility delivery in LiST. Due to high rate of facility-based delivery in Rwanda, it is assumed that SBA and facility-based delivery are nearly the same

†Exclusive breastfeeding data are from national DHS

no subnational or rural data are available

## RESULTS

### Comparison of endline coverage estimates

**Figure 1.**
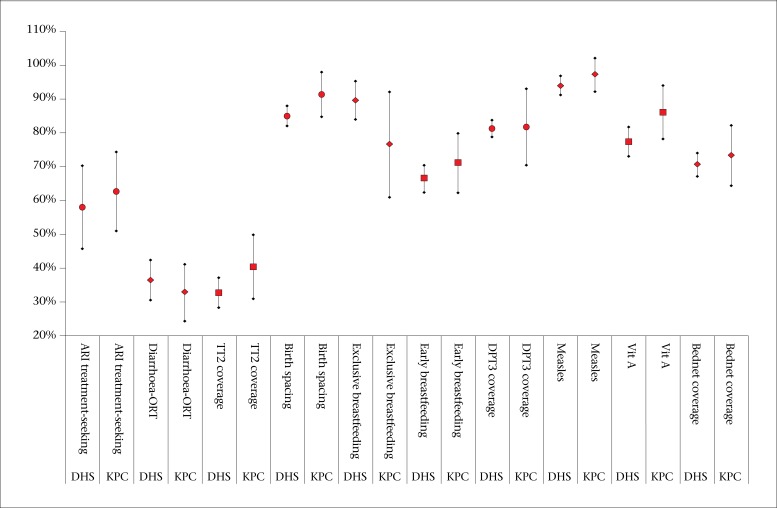
Comparison of overlapping KPC and RDHS indicators

The KPC and RDHS surveys produced comparable estimates for 10 out of 15 indicators: treatment-seeking for ARI, use of ORS, tetanus coverage in pregnancy, adequate birth spacing, exclusive breastfeeding, early initiation of breastfeeding, DPT3 coverage, measles vaccine coverage, vitamin A supplementation coverage, and bednet coverage (Table 4). Figure 1 shows the relationships between the means and the confidence intervals for those indicators found to be consistent.

**Figure 2.**
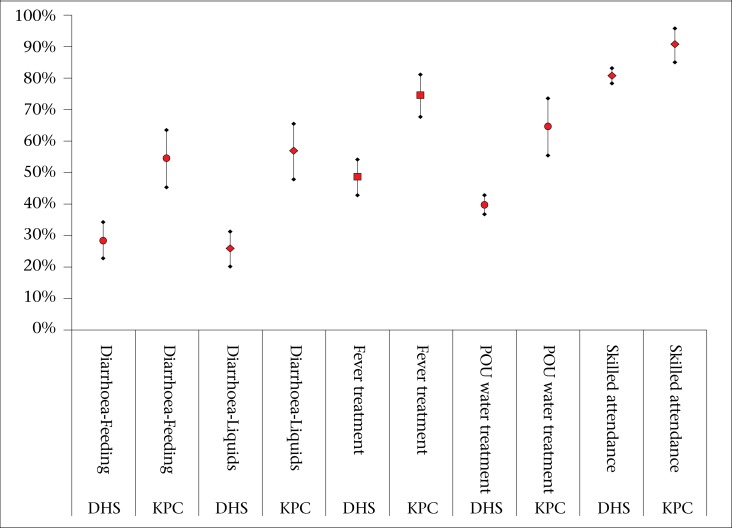
Comparison of divergent KPC and RDHS indicators

For five indicators—treatment-seeking for fever, feeding during diarrhoea, liquids during diarrhoea, point-of-use water treatment, and skilled delivery attendance—the two surveys produced significantly different results. The scale and trend of these differences is shown in Figure 2. These differences may reflect methodological inconsistency or actual differences in the population means. The latter interpretation is supported by the fact that the KPC data were collected three to nine months after the DHS; the last year of the Kabeho Mwana Project was a period of national and project-level acceleration in implementation, and all five KPC indicators provided higher estimates than the DHS.

### Comparison of mortality estimates

The mortality rate estimated by LiST is nearly identical to the mortality rate measured by the re-analyzed RDHS (Table 5). When area-specific baseline mortality data of the project were used from the re-analyzed RDHS to model-estimated mortality, based on changes in KPC data, LiST estimated 2011 under-five mortality to be 83 per 1,000 livebirths. The 2011 RDHS-measured under-five mortality was 83 per 1,000 livebirths.

**Table 3.** Comparison of indicator definitions in RDHS and KPC

Name	Indicator definition	Difference between KPC and RDHS in the way the questions were asked	Sampling difference
Treatment for malaria or fever	% of children aged below 5 years with a febrile episode during the last two weeks, who were treated with an effective antimalarial drug within 24 hours after the fever began	RDTs have been instituted, eliminating presumptive treatment; comparison of the indicators during the transition period impossible	[1]
Treatment-seeking for fever*	% children aged below 5 years with fever in the past 2 weeks, who were taken to an appropriately-trained care provider	Treatment for fever and ARI symptoms were asked simultaneously in the DHS while, in the KPC treatment, were asked separately for each condition	[1]
ARI antibiotic treatment*	% children aged below 5 years with cough and respiratory difficulty or rapid breathing in the past 2 weeks, who were treated with antibiotics	Treatment for fever and ARI symptoms were asked simultaneously in the DHS while, in the KPC treatment, were asked separately for each condition	[1]
Liquids during diarrhoeal episode*	% children aged below 5 years with diarrhoea whose caregivers offered more liquid than usual to the child	Sick child-feeding questions were asked at the beginning of the interview before asking the specifics of the condition	[1]
Feeding during diarrhoea*	% children aged below 5 years with diarrhoea whose caregivers offered the same or more food than usual to the child	Sick child-feeding questions were asked at the beginning of the interview before asking the specifics of the condition	[1]
ORT-use*	% children aged below 5 years with diarrhoea in the past 2 weeks, who received oral rehydration therapy or recommended home solution		[1]
Zinc-use	% children aged below 5 years with diarrhoea in the past 2 weeks, who received zinc treatment	Not asked in the DHS	
Tetanus toxoid coverage*	% of mothers with children aged 0-23 month(s), who received at least 2 tetanus toxoid vaccines before the birth of their youngest child		
Skilled delivery assistance*	% of last-born living children aged 0-23 month(s) whose births were attended by skilled personnel		[2]
Postnatal check-up	% of last-born children aged 0-23 month(s), who received a postnatal visit from an appropriately-trained health worker within 3 days after birth	DHS only asked about postnatal check for non-facility deliveries; KPC did not distinguish between check for child or for mother	[3]
Birth spacing*	% of last-born children aged 0-23 month(s), who were born at least 24 months after the previous surviving child		
Exclusive breastfeeding*	% of children aged 0-5 month(s), who were exclusively breastfed during the last 24 hours	KPC asked only if child was taking any other fluids or any foods. DHS asked about a list of specific foods and beverages	
Early initiation of breastfeeding*	% of mothers of children aged 0-23 month(s), who initiated breastfeeding within 1 hour of the last birth		
DPT3 coverage*	% of children aged 12-23 months, who had received a DPT3 vaccination prior to the survey as documented on the vaccination card		[3]
Measles vaccination coverage*	% of children aged 12-23 months, who received a measles vaccination		[3]
Vitamin A coverage*	% of children aged 6-23 months, who received high-dose vitamin A supplementation within the last six months		[3]
Bednet coverage*	% children aged 0-23 month(s), who slept under a treated bednet the previous night as reported by the caregivers		[3]
Handwashing	% of children aged 0-23 month(s), whose caregivers can cite a designated site for handwashing, show soap at that site, and who wash their hands after using the toilet on at least one other key occasion	KPC asked for handwashing place, did not require water, DHS did not ask about behaviour	[4]
Point-of-use (POU) water treatment*	% of households of children aged 0-23 month(s) that treat water effectively (chlorine, boiling, or filtering)		[5]

*Included in analysis

[1] KPC targeted households with children who had been ill in the last 2 weeks

[2] KPC asked about the child, DHS asked about the delivery

[3] DHS included all children in a household, KPC included only one randomly selected

[4] DHS sampled all households, KPC only sampled households with children aged below 24 months

[5] DHS sampled all households, KPC only sampled households with a child aged below 24 months

**Table 4.** Comparison of coverage estimates from the KPC and RDHS for the Kabeho Mwana Project area

Outcome indicator	RDHS 2010			KPC 2011			Significant difference (p<0.05)
	No.	Mean%	95% CI	No.	Mean%	95% CI	
Treatment-seeking for fever	337	48.7	(43.1-54.3)	226	74.6	(68.0-81.3)	yes
Treatment-seeking for ARI	86	57.9	(45.6-70.1)	115	62.7	(51.3-74.0)	no
Feeding during diarrhoeal episode	312	28.7	(23.0-34.4)	167	54.6	(45.7-63.6)	yes
Liquids during diarrhoeal episode	312	25.9	(20.2-31.5)	167	56.9	(48.0-65.8)	yes
Use of ORT	312	36.6	(30.7-42.4)	167	32.8	(24.5-41.2)	no
TT2 coverage	701	32.9	(28.5-37.3)	120	40.5	(31.1-49.9)	no
Skilled delivery attendance	729	78.7	(75.2-82.4)	120	90.7	(85.4-96.0)	yes
Birth spacing	517	84.6	(81.8-87.5)	77	91.0	(84.4-97.6)	no
Exclusive breastfeeding	169	89.2	(83.7-94.7)	35	76.4	(61.0-91.8)	no
Early initiation of breastfeeding	701	66.3	(62.3-70.2)	120	70.9	(62.2-79.6)	no
DPT3 coverage (on health card)	1,616	81.4	(78.5-83.4)	53	81.4	(70.2-92.6)	no
Measles vaccine coverage	335	93.6	(90.9-96.3)	62	96.8	(92.0-101.6)	no
Vitamin A coverage	523	77.1	(72.9-81.4)	85	85.8	(77.9-93.6)	no
Bednet coverage	698	70.5	(66.9-73.8)	120	73.0	(64.3-81.7)	no
Point-of-use (POU) water treatment	2,491	39.9	(36.8-43.0)	120	64.7	(55.6-73.7)	yes

The number of observations shown here for comparison is the unweighted sample-size for the KPC and the weighted sample-size for the RDHS (DHS weighting produces an adjusted figure very close to the actual sample-size)

**Table 5.** Comparison of mortality estimates from the RDHS and the LiST model for Kabeho Mwana districts

Year	Under-five mortality rate (per 1,000 livebirths)	
	LiST RDHS re-analysis model	Mortality measured by RDHS re-analysis
2007	110	110
2011	83	83

## DISCUSSION

We draw three main lessons from this study and our experience. These are described below.

### Lesson 1: The reliability of local estimates provided through small population-based scientific surveys, like the KPC, is demonstrated again

Our findings strongly support the comparability of the KPC survey with the DHS, with 10 out of 15 key coverage indicators having estimates comparable to the RDHS. The consistency of the results of the LiST model when the KPC results are entered further validates the KPC methodology and corroborates previous findings that local surveys can be used in estimating mortality and lives saved as a result of public health interventions (10,20,21).

We have also identified cases of lack of congruence between KPC and RDHS measures. For 5 out of 15 indicators, we found significant differences between the KPC and the RDHS. We identified three possible reasons for these differences (only one related to the sampling approach): (i) the results may reflect actual changes in coverage in the three-to nine-month interval between the two surveys; (ii) it is possible that the KPC (because it did not rely on cluster sampling) might be more accurate, especially for the disease-specific indicators; and (iii) the role of the KPC data-collectors in relation to the project as interviewers who were the project and MOH staff could also have resulted in biases both of expectation and of social desirability. Whatever the cause is, it is impossible to say which of the two surveys more accurately reflects the true population averages. Their proximity bolsters the validity of both methodologies.

### Lesson 2: Survey work remains challenging and requires attention to quality, regardless of the scale of the exercise

Some of the wrong assumptions about small population-based surveys come from a lack of distinction between ‘rapid’ knowledge surveys carried out ad-hoc without respect for appropriate sampling and research guidelines and scientific surveys, such as the KPC. We have specifically and only discussed the value of small population-based surveys following proper statistical sampling rules and respecting standards for the quality of collection, processing, and analysis of data. While implementation of the KPC took less time and produced results more rapidly than the more ambitious RDHS, it was far from ‘rapid’ or careless in its design and supervision.

The demonstrated validity of this particular KPC survey does not eliminate all concerns about the quality of surveys done without the rigorous central control and technical oversight, which characterizes the DHS (6). Rather, our study indicates that, with proper caution, respect for appropriate standards, and supervision (1,9), the risk can be mitigated.

Practitioners often forget that non-sampling errors are equally treated or are more frequent and critical than sampling errors in affecting the validity of survey estimates. The non-sampling errors which may have biased the estimates for some of the KPC indicators could be addressed by contracting with research firms, survey groups, or universities, rather than the implementing staff. However, this approach could have downsides in the lost opportunity to build capacity of staff and MOH and ownership of the data. In the case of Kabeho Mwana, however, training of data-collectors seems to have averted theoretical risk of a survey or response bias. Whatever approach is selected, it will be needed to balance available resources and costs against the real-world needs of managers and decision-makers.

### Lesson 3: Cost needs to be considered in line with the appropriateness of the survey to the management questions

The KPC survey used in this study costs approximately US$ 25,400 to cover six districts in three regions and was representative of the entire estimated target population of 1.8 million. This is consistent with previous estimates that a one-district or one-area KPC survey will cost from US$ 15,000 to 25,000 in most countries (1). In contrast, a DHS survey costs upward of the quarter of a million dollars for obviously very different benefits.

These broad comparisons are, however, difficult given that KPCs can be tailored to specific programme questions and targeted health indicators while the DHS provides breadth and depth of information to a wide national and global audience.

The real question is what information is acquired at what level. Sometimes, efforts are made to provide lower-level disaggregation of estimates for DHS surveys but this has very substantial cost implications. From a district management perspective, specific efforts to improve community health will require action on timeframes unrelated to national data-collection efforts.

The value of the KPC is ultimately rooted in its potential for providing local information to local actors, below regional levels, within a specific timeframe, which may not fit that of a national survey, even if it were to go to the expense of providing district estimates. In the context of the district system strengthening, shared national ownership, and decentralization, these are not marginal issues.

### Reliability of KPC data

The KPC is demonstrated, in a real-world example, as a reliable tool to obtain subregional point estimates of health coverage and trends comparable to those obtained using the DHS methodology. It is a powerful tool for evidence-based programming, and, in a global context of increased demand for decentralization, accountability, and local ownership, local, national and global health practitioners should challenge their assumptions and consider why it remains underused. The following are the assumptions:

- Concerns for quality of the data are legitimate but we provide one more among many examples of appropriate and cost-effective use of the method to produce reliable coverage indicators at the population level. Moving the discussion from rejection of small surveys towards efforts to strengthen their more systematic use when appropriate and the continued promotion of best practices to ensure quality, would be a major step forward.
- There has not been, to our knowledge, a sound study for the cost of these surveys, and the cost-benefit of informing local district managers in progress towards population-based targets across a full range of primary care services. Neither is there evidence, however, for the frequent jump to the misled conclusion that “population surveys are too expensive.” We would argue that the costs we report on would be reasonable for a large number of nationally- or internationally-funded programmes at the district level—the devil is in moving from assumptions to specifics. District managers strongly value this basis of information when it is provided to them (22,23).
- We stayed clear of contrasting population-based survey data with routine services data. The assumptions that population surveys are expensive or unreliable combine with the need to provide information to decision-maker to make a case for investing in and relying on routine health information systems. Absent from this argument are the facts that: investing has a cost in time and funds, information systems have their own quality and reliability issues, and, last but not the least, provide substantially different information, most notably, entirely missing those who do not access services due to poverty or other forms of exclusion. Our objective is not to oppose those two sources of information but to stress that each source has its value added and that the balance of value-to-cost for small population-based surveys is underestimated due to assumptions that are not based on evidence.

### Conclusions

Small population-based surveys, such as KPC, should be used more readily by subnational programmes to meet their specific needs. The validity and reliability and reasonable cost of small population-based surveys beg for their more frequent use to produce, at minimum, regular pre-post data to the managers of decentralized health systems and local project interventions.

## ACKNOWLEDGEMENTS

We would like to thank the international NGOs Concern Worldwide, the International Rescue Committee, and World Relief and their staff who implemented the Kabeho Mwana Project, particularly Rose Luz, Project Director and all the project staff in Rwanda, who completed the endline survey. Jennifer Weiss of Concern Worldwide, Justine Landegger, and Paul Amendola of the International Rescue Committee, and Melanie Morrow and Melene Kabadege of World Relief provided indispensable support for the project and the survey and reviewed this manuscript.

We would like to thank colleagues at ICF International for their contributions, including Clara Burgert who provided recoded district data for the Rwanda DHS; Alex Izmukhambetov who did the mortality-related calculations and Tom Pullum who provided guidance on the statistical analysis. We would also like to acknowledge the invaluable contributions of Jim Ricca and Bill Weiss for their thorough review and comments on this manuscript, Laban Tsuma for his guidance, and Natasha Wad for her help with preparation and formatting of tables and citations.

**Conflict of interest:** Authors declare no competing interests.
